# Superconducting parity effect across the Anderson limit

**DOI:** 10.1038/ncomms14549

**Published:** 2017-02-27

**Authors:** Sergio Vlaic, Stéphane Pons, Tianzhen Zhang, Alexandre Assouline, Alexandre Zimmers, Christophe David, Guillemin Rodary, Jean-Christophe Girard, Dimitri Roditchev, Hervé Aubin

**Affiliations:** 1 ^1^LPEM, ESPCI Paris, PSL Research University, CNRS, Sorbonne Universités, UPMC University of Paris 6, 10 rue Vauquelin, Paris F-75005, France; 2 Centre de Nanosciences et de Nanotechnologies, CNRS, Univ. Paris-Sud, Universités Paris-Saclay, C2N–Marcoussis, Marcoussis 91460, France

## Abstract

How small can superconductors be? For isolated nanoparticles subject to quantum size effects, P.W. Anderson in 1959 conjectured that superconductivity could only exist when the electronic level spacing *δ* is smaller than the superconducting gap energy Δ. Here we report a scanning tunnelling spectroscopy study of superconducting lead (Pb) nanocrystals grown on the (110) surface of InAs. We find that for nanocrystals of lateral size smaller than the Fermi wavelength of the 2D electron gas at the surface of InAs, the electronic transmission of the interface is weak; this leads to Coulomb blockade and enables the extraction of electron addition energy of the nanocrystals. For large nanocrystals, the addition energy displays superconducting parity effect, a direct consequence of Cooper pairing. Studying this parity effect as a function of nanocrystal volume, we find the suppression of Cooper pairing when the mean electronic level spacing overcomes the superconducting gap energy, thus demonstrating unambiguously the validity of the Anderson criterion.

## Abstract (web summary)

How quantum size effects affect superconductivity has been predicted, but it has never been verified. Here, Vlaic *et al*. report superconducting parity effect as a function of lead nanocrystal volume, unambiguously validating the Anderson criterion.

The addition energy of an electron to a superconducting island, weakly coupled to the environment by the capacitance *C*_Σ_, is given by (see Methods):


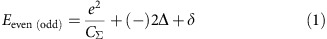


where the first term is the Coulomb energy, the second term depends on the parity of electron occupation number as a consequence of the formation of a Cooper pair^1^,^2^ and the third term is the electronic level spacing in the island. This parity effect has been observed in large: 1 μm micro-fabricated Al islands, through direct measurement of the charge capacitance of the island^2^, through the even–odd modulation of the addition energy in single-electron transistors^3^,^4^,^5^,^6^ or the parity dependence of the Josephson current in Cooper pair transistors^7^,^8^,^9^.

Until now, the parity effect on the addition energy has never been observed in small nanocrystals (NCs) near the Anderson limit^10^, reached at a volume about *V*_Anderson_≃100 nm^3^, where the mean electronic level spacing <*δ*> equals the superconducting gap energy Δ.

In single-electron transistors fabricated with nanosized superconducting grains of aluminium^11^,^12^, the 2*e* modulation of the addition energy could not be observed directly. Also, because only a few devices could be fabricated, testing the Anderson criterion was not possible with this approach. Indirect indications for the disappearance of superconductivity in small superconducting grains came from magnetization measurements^13^,^14^; because these measurements were averaged over macroscopic quantities of NCs, the link to the Anderson limit remained ambiguous.

In this work, we present a new system that enables a study of single and isolated NCs across the Anderson limit, where the NCs can be reproducibly obtained in large quantities. The superconducting gap energy and the transition temperature are measured through a study of the superconducting parity effect in the addition energy of the NCs. This constitutes an alternative approach to conventional tunnelling measurement of the superconducting gap in the quasiparticle excitation spectrum, which cannot be accessed as a consequence of the Coulomb gap at zero bias.

## Results

### Sample preparation

The observation of the parity effect is challenging as it requires clean systems, free of impurity states responsible for the so-called quasiparticle poisoning^9^,^15^. Furthermore, scanning tunnelling spectroscopy of isolated NCs requires, in addition to the tip–NC tunnel barrier, a second tunnel barrier between the NC and the conducting substrate^16^,^17^, as sketched in Supplementary Fig. 1.

**Figure 1:**
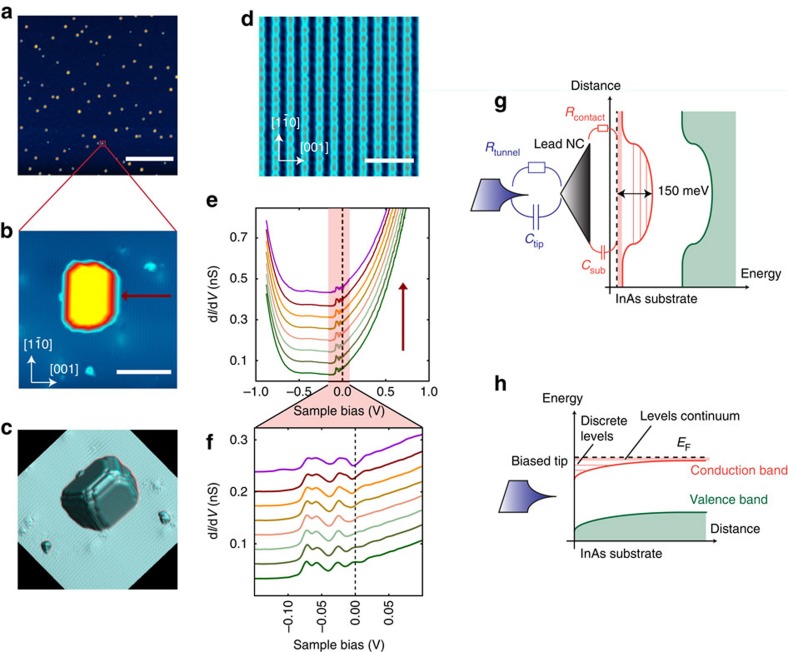
Pb NCs on InAs (110). (**a**) 1 μm × 1 μm topographic STM image (1 V, 30 pA) of Pb NCs grown on the (110) InAs surface of sample A. Scale bar, 300 nm. (**b**) Zoom on 30 nm × 30 nm area, showing a Pb NC. Scale bar, 10 nm. (**c**) 3D Laplacian Δ_*x*y_*z*(*x*,*y*) image of a NC. (**d**) 6.5 nm × 6.5 nm atomic resolution image of InAs (110) obtained near the NC. Scale bar, 2 nm. (**e**) DC measured at several distances from the Pb NC along the red arrow in **b**. (**f**) Zoom at low bias showing the conductance peaks due the discrete levels of the tip-induced quantum dot. (**g**) Sketch of the band bending below the Pb NC due to the pinning of the Fermi level at the charge neutrality level. (**h**) Sketch of the band bending induced by the tip leading to the formation of a quantum dot.

In this work, the Pb NCs are obtained by thermal evaporation of a nominal 0.3 monolayer of Pb on the (110) surface of InAs heated at *T*=150 °C. The (110) surface is obtained by cleaving an n-type InAs substrate in ultra-high vacuum at a base pressure *P*∼10^−10^ mbar. Two distinct samples (A and B) have been prepared with slightly different NC concentrations and sizes. The volume of the NCs ranges from 20 nm^3^≃0.2 *V*_Anderson_ to 800 nm^3^≃8*V*_Anderson_ while the height ranges from 1 unit cell (0.495 nm) to 5.2 nm, see Supplementary Fig. 2 and Supplementary Note 1 for details on NC volume determination. The scanning tunnelling microscopy (STM) topographic images (Fig. 1a–c; Supplementary Fig. 3) for sample A and sample B, respectively, show that Pb grows in the Volmer–Weber, that is, Island mode^18^. The three dimensional Laplacian image Δ_*xy*_*z*(*x*,*y*) (Fig. 1c) shows that the NCs are well crystallized and expose mostly the (111) planes of the cubic face-centred Pb structure, as indicated by the observation of the characteristic hexagonal shape of the (111) facets. Surrounding these NCs, the surface remains free from adsorbate, as atomic resolution images of the (110) InAs surface prove (Fig. 1d).

### Tip-induced QDot on the InAs surface

Figure 1e shows the differential conductance (DC) d*I*/d*V* measured on the InAs surface at several distances, from 0 to 10 nm, of a Pb NC. The data are measured at *T*=1.3 K, unless indicated otherwise, using a standard lock-in procedure (see Methods). The data indicate that the Fermi level is in the conduction band of InAs as expected for this n-doped sample. With a sulphur dopant concentration, ND∼6 × 10^16^ cm^−3^, the Fermi level is 21 meV above the conduction band minimum. A zoom on these spectra (Fig. 1f) shows multiple peaks that result from the discrete levels of the tip-induced quantum dot (QDot), a phenomena that has also been observed in previous works^19^. This demonstrates that Pb deposition on InAs do not produce any significant defects and doping. Indeed, in the presence of defects or adsorbate, the surface of III–V semiconductors present interface states that pin the Fermi level at the charge neutrality level^20^,^21^ (Fig. 1g). For InAs, this level is located 150 meV above its conduction band minimum, which leads to the formation of an electron accumulation layer as shown by numerous photoemission experiments^20^,^22^. In contrast, perfectly clean (110) surfaces do not present any interface states and consequently the Fermi level is not pinned. Thus, the electric field from the STM tip can easily shift the conduction band and generates the so-called tip-induced QDot^19^, as sketched in Fig. 1h. While the energy of the QDot levels can shift on long distances, see Supplementary Fig. 4, as a consequence of variations in the electrostatic environment due to the random distribution of Pb NCs and sulphur dopants, we see (Fig. 1f) that the QDdot levels are not altered on short distances (<10 nm) near the NCs. Only a weak broadening of the QDot levels is observed, likely a consequence of their weak tunnel coupling with the Pb NCs.

### Coulomb blockade and nature of the tunnel barrier

**Figure 2:**
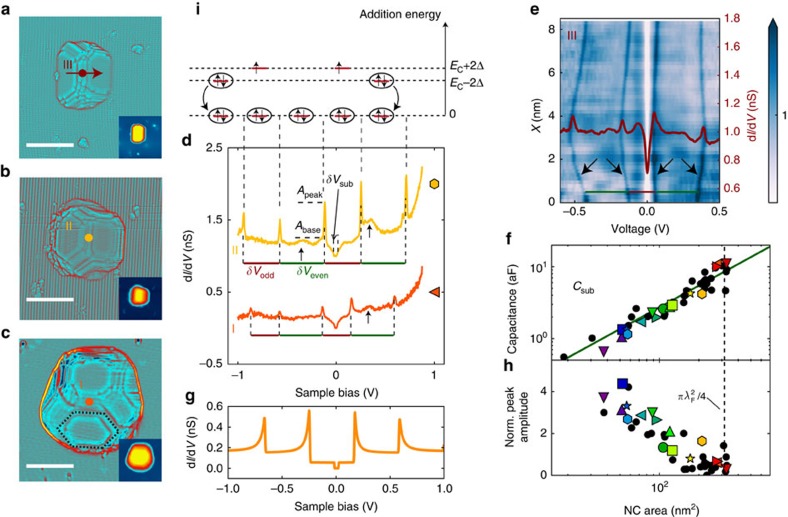
Pb NCs in the regime of Coulomb blockade. (**a**–**c**) 30 nm × 30 nm Laplacian Δ_*xy*_*z*(*x*,*y*) topographic images (30 pA, 1 V) of NCs of decreasing size, labelled I to III, where the hexagonal shape of the (111) facets is visible, as shown by the dash line in **c**. The scale bars correspond to 10 nm. The insets show the corresponding topographic STM images. (**d**) DC measured at the centre of NC I and II, indicated by dots in **b**,**c**. The addition voltages *δV*_odd_ and *δV*_even_ can be identified for each curve. The black arrows indicate the local maxima in the density of states due to quantum well states. The coloured symbols identify the corresponding data points in **f**,**h**, and Fig. 5. (**e**) DC map as function of sample bias and distance measured on NC III along the red arrow shown in **a**. The black arrows indicate the Coulomb peak lines. (**f**) Capacitance *C*_sub_ extracted from the Coulomb gap at zero bias. It scales linearly with the NC area. (**g**) Simulation of the DC for NC II using the weak coupling model^26^. (**h**) Normalized Coulomb peak amplitude *A*_norm_=(*A*_peak_–*A*_base_)/*A*_base_, this value decreases at the approach of the area *π*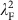/4. (**i**) Sketch of electron occupation of NC II.

**Figure 3:**
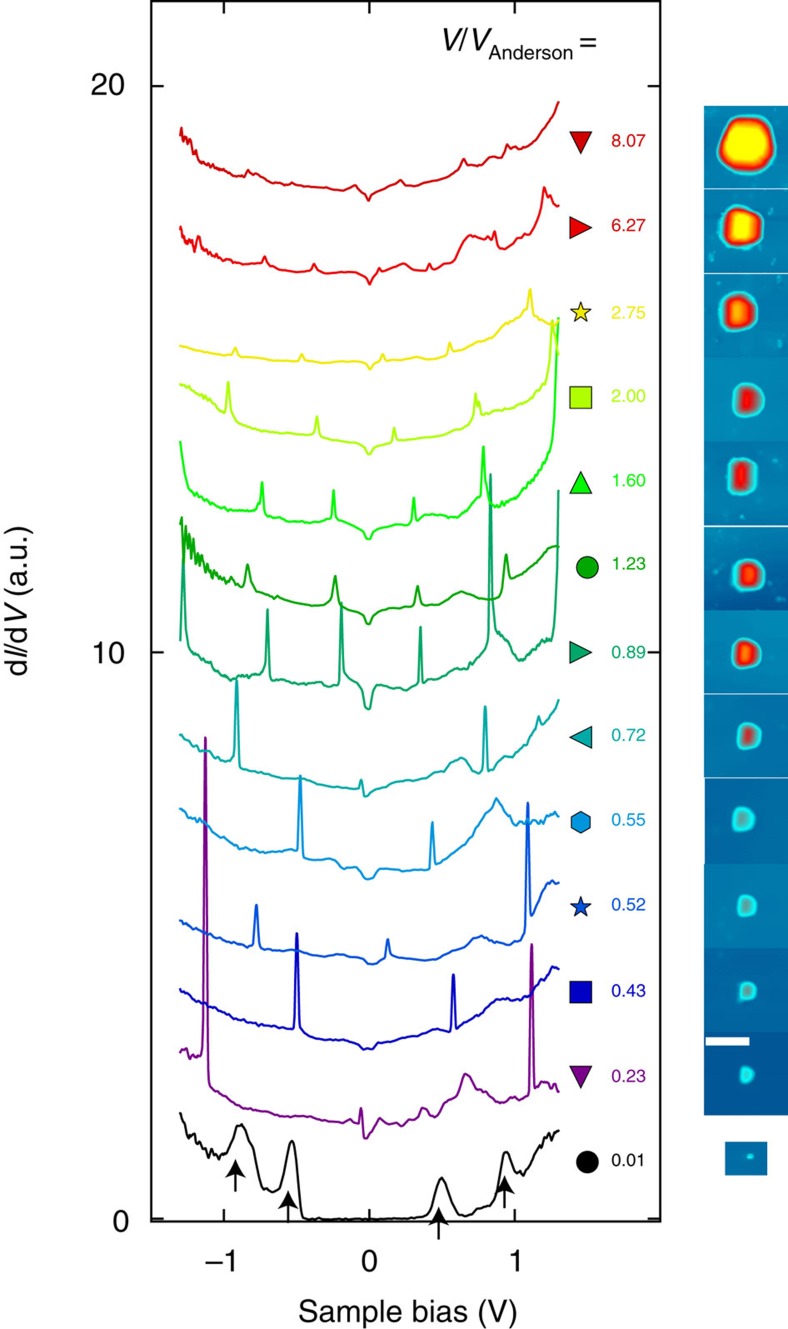
DCs for increasing NC volume. The coloured symbols identify the corresponding data points in Figs 2f,h and 5. For each spectrum, the corresponding NC and the volume ratio *V*/*V*_Anderson_ are shown on the right. Note that for the smallest NC (bottom black curve) no Coulomb peaks are observed, instead a large Coulomb gap and broad quantum well peaks are observed.

On NCs of three distinct sizes shown in Fig. 2a–c, representative DC spectra are shown in Fig. 2d,e. They display a Coulomb gap at zero bias of width *δV*_sub_=*e*/(*C*_sub_+*C*_tip_), where *C*_sub_ (*C*_tip_) is the capacitance between the NC and the substrate (tip). The data also display sharp Coulomb peaks where the voltage interval between the peaks provides the addition voltage *δV*_add_ for an electron, which is related to the addition energy by: *δV*_add_=*E*_add_/*eη*, where 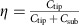 is the arm lever; see the Methods section for a derivation of these relations. Furthermore, the DCs may also display broad additional peaks, of weak amplitude in large NCs, *V*/*V*_Anderson_>1, as indicated by arrows in Fig. 2d, but of large amplitude in small NCs, *V*/*V*_Anderson_<<1, as indicated by arrows in Fig. 3. These broad peaks are the signature of quantum well states in the Pb NCs due to strong confinement in the <111> direction as observed in scanning tunnelling studies of thin layers of Pb^23^.

The colour map in Fig. 2e shows that *δV*_add_ changes slightly with the tip position above the NC, as consequence of the variation in the tip–NC capacitance *C*_tip_. Figure 3 shows the DCs for 13 additional NCs, from which the capacitance *C*_sub_ is extracted and shown as coloured symbols in Fig. 2f and Supplementary Fig. 5. On these last plots, data points shown as black circles of 24 other NCs are also included, for which the DCs are not shown. Figure 2f shows that *C*_sub_ increases linearly with the area *A* as *C*_sub_=*Aɛ*/*d*, using *ɛ*=12.3, the dielectric constant of InAs and *d*=4 nm for the effective tunnel barrier thickness.

As no dielectric insulator has been deposited on the surface and no Schottky barrier exists at metal–InAs interfaces^21^,^22^, the origin of the tunnel barrier and the meaning of the thickness *d* appear clearly only after one realizes that the Fermi wavelength of the two-dimensional (2D) gas in InAs is larger than the lateral size of the NCs. At the interface between the Pb NC and InAs, the Fermi energy in InAs is at the charge neutrality level, *E*_F_=150 meV (refs 21, 22), which gives for the Fermi wavelength *λ*_F_=20 nm. As known from numerous works with quantum point-contacts formed in 2D electron gas^24^,^25^, the transmission coefficient *T* decreases for constrictions smaller than the Fermi wavelength. Because a NC covers only a fraction of the area ≃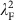, its transmission coefficient with the 2D gas is significantly smaller than one, which explains the observation of the Coulomb blockade. For a small NC, the weak coupling model^26^ can be used to describe the data, as shown in Fig. 2g. This model shows that the contact impedance is of the order of *R*_contact_∼10 MΩ, implying that the transmission coefficient *T*=*R*_contact_*e*^2^/*h*=0.0025 is weak as anticipated. In this model, the magnitude of the Coulomb peaks increases with the ratio *R*_tunnel_/*R*_contact_, as observed on the DC curves measured as function of tip height (Supplementary Fig. 6). Figure 2h shows the amplitude of the Coulomb peak, normalized to its base value, as function of NC area. The amplitude is constant for small area (<100 nm^2^) but decreases quickly for area approaching *π*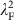/4≃300 nm^2^. This behaviour cannot be described by the weak coupling model just discussed; however, it can be understood by considering models of Coulomb blockade in the strong coupling regime^27^,^28^. These models show that the Coulomb oscillations disappear when *T* approaches unity, when charge fluctuations between the NC and the substrate become significant. Figure 3 shows that the Coulomb peaks of the largest NCs have almost completely disappeared. The fact that the amplitude of the Coulomb peaks decreases for NCs area approaching 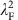 confirms our interpretation that the tunnel barrier is due to a quantum constriction of the electronic wave function at the interface between the NC and the 2D gas. Thus, the dielectric thickness *d*=4 nm extracted from *C*_sub_ above is actually set by the Debye length of the 2D gas and *C*_sub_ actually corresponds to the quantum capacitance of InAs.

### Superconducting parity effect

**Figure 4:**
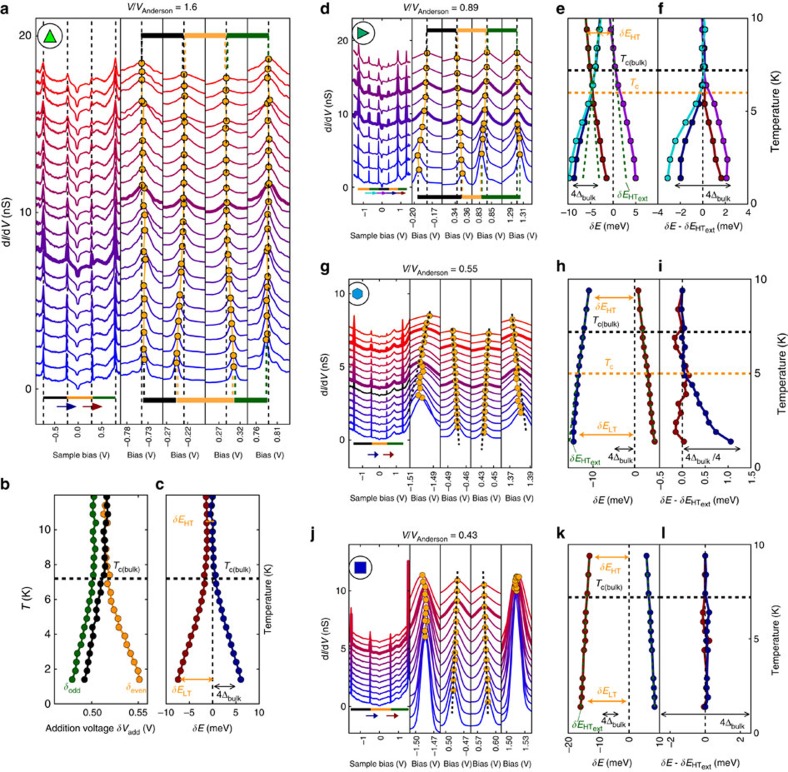
Parity effect as function of temperature. DC and addition energies for four NCs of decreasing volume, where *V*/*V*_Anderson_ is indicated on top of the panels. (**a**,**d**,**g**,**j**) DC curves as function of temperature. The voltage separation between the Coulomb peaks, that is, the addition voltage, is indicated by the horizontal bars of different colours. In the same panels, zoom on the Coulomb peaks are shown where the maxima are indicated by orange dots. For **a**, the addition voltages are plotted as the function of temperature in **b** with corresponding colours. The coloured symbols (top left of panels) identify the corresponding data points in Figs 2f,h and 5. (**c**,**e**,**h**,**k**) Difference in addition energies between two charge configurations given by 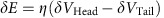, where the head (tail) refers to the arrows shown in the corresponding panels. (**f**,**i**,**l**) Difference 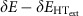, where the dash green line 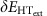, is obtained from the extrapolation of *δE* at high temperature. For **b**,**c**,**e**,**f**,**h**,**i**,**k**,**l**, the value *T*_c_ (bulk) is indicated as a black dash line. The extracted *T*_c_ is shown as orange dash lines. A double-headed arrow provides the scale for the energy gap 4Δ_bulk_ of bulk Pb.

Owing to this highly clean type of tunnel junction, free from quasiparticle poisoning, the superconducting parity effect in the NCs can be observed through the even–odd modulation of the addition voltage, as shown in Figs 2d,e, 3 and 4. The addition voltages can be precisely extracted due to the sharpness of the Coulomb peaks, which voltage positions are obtained through a fit with a Lorentz function (Supplementary Fig. 7). As sketched in Fig. 2i and shown by equation (1), the addition voltage *δV*_even_ for injecting an electron in an even parity NC is higher than *δV*_odd_ for injecting an electron in an odd parity NC, where the energy difference is given by the binding energy of the Cooper pair. Figure 4a shows the DCs for a large NC, *V*/*V*_Anderson_=1.6, as function of temperature. The corresponding addition voltages, shown in Fig. 4b, are almost equal above *T*_c_=7.2 K, the superconducting transition temperature of bulk Pb. However, an even–odd modulation is observed at low temperature *T*=1.3 K. The difference in the addition energies between two successive charge configurations is obtained from *δE*=*eη*(*δV*_even_–*δV*_odd_). For this large NC, four Coulomb peaks are observed, which provide three distinct addition voltages indicated by the horizontal bars. From these addition voltages, two distinct values of the addition energy difference *δE* between two charge configurations are obtained and given by *δE*=*η*(*δV*_Head_−*δV*_Tail_), where the head (tail) refers to the coloured arrows in the panel. These two values of *δE* are shown in Fig. 4c as the function of temperature. Their values are near zero at high temperature, *δE*_HT_∼0, and increase below *T*_c_=7.2 K to reach, at low temperature, the theoretically expected value |*δE*_LT_|∼4Δ_bulk_ (ref. 1), where Δ_bulk_=1.29 meV is the superconducting gap of bulk Pb. The value *δE*_LT_ changes sign as one goes from the difference between two addition energies *δE*=*eη*(*δV*_even_−*δV*_odd_) to the next difference *δE*=*eη*(*δV*_odd_–*δV*_even_).

**Figure 5:**
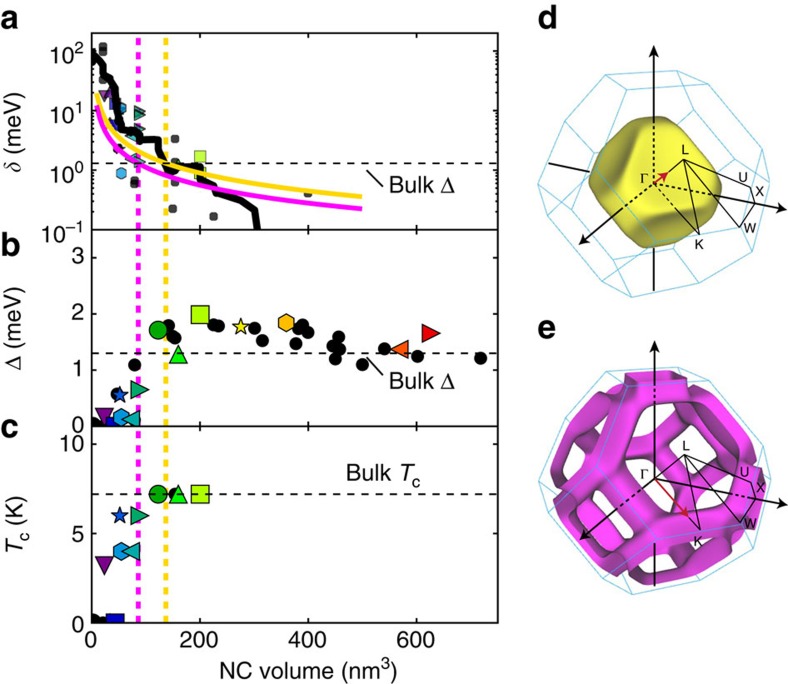
Across the Anderson limit. (**a**) Level spacing extracted from the addition energies measured above *T*_c_. The experimental data (symbols) are highly scattered as a consequence of the random electronic level distribution. However, the average level spacing, shown by the smoothed black line, is of the order of magnitude of the calculated theoretical values shown as coloured lines. The horizontal dash line indicates the bulk superconducting energy gap. (**b**) Superconducting gap Δ extracted from the difference in addition energies between high and low temperature. The horizontal dash line indicates the bulk superconducting energy gap. (**c**) Transition temperature as the function of NC volume. The horizontal dash line indicates the bulk transition temperature *T*_c_=7.2 K. For all panels, the two vertical dash lines indicate the volumes where the level spacing reaches the superconducting energy gap at the wavevectors shown by red arrows on the two Fermi surfaces on the right. The coloured symbols identify the corresponding DC curves in the other figures. For the black circles, the DCs are not shown. (**d**) Fermi surface (FS1) of the hole-type band of Pb. (**e**) Fermi surface (FS2) of the electron-type band of Pb.

For NCs smaller than the Anderson volume (Fig. 4d,g,j), we observe that *δE*_HT_ is non-zero, which indicates that the electronic level spacing *δ* has now a significant contribution to the addition energy, following equation (1). The values of *δE*_HT_ are distinct between successive charge configurations. Indeed, in metallic systems, the electronic levels are randomly distributed as described by random matrix theory (RMT)^29^. Collecting the values *δE*_HT_ for all NCs, Fig. 5a shows that, in average, the evolution of *δE*_HT_ with NC volume can be properly described by the relation:


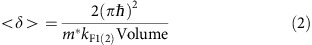


using m*=1.2 *m*_e_ for the effective mass, where *k*_F1_=7.01 nm^−1^ and *k*_F2_=11.21 nm^−1^ are characteristic wavevectors of the two Fermi surfaces FS1 and FS2 of Pb.

For the NC of volume *V*/*V*_Anderson_=0.89 (Fig. 4d), while the level spacing *δE*_HT_ is large, the shift of the Coulomb peaks due to the parity effect is still dominating the temperature dependence and can be observed directly on the raw data and the addition energy difference *δE* plotted as the function of temperature in Fig. 4e. A line 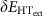 is extrapolated from high temperature and the difference 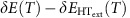 gives the temperature dependence of the superconducting gap (Fig. 4f), which shows that the critical temperature *T*_c_≃6 K is smaller than the bulk value. The amplitude of the superconducting gap is obtained from 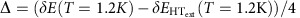. For this NC, the superconducting energy gap is about two times smaller than the bulk value, Δ=Δ_bulk_/2.

For the smaller NC of volume *V*/*V*_Anderson_=0.55 (Fig. 4g), the level spacing *δE*_HT_ is larger and has a temperature dependence that dominates the shift of the Coulomb peaks with temperature. This shift could be the consequence of thermally induced electrochemical shifts or temperature-dependent strain or electric field effects. While the parity effect is barely visible on the raw data, using the procedure employed for the previous NC, the temperature *T*_c_≃5 K value and the energy gap Δ≃Δ_bulk_/4 can be extracted (Fig. 4i).

Finally, for the smallest NCs *V*/*V*_Anderson_=0.43 (0.34), shown, respectively, in Fig. 4j and Supplementary Fig. 8h, they have the largest level spacing *δE*_HT_ and, even though the addition energies are measured with much higher resolution than the superconducting gap energy, no parity effect can be observed in Fig. 4l and Supplementary Fig. 8j, respectively.

For 13 NCs where the DCs have been acquired as the function of temperature, some of which are shown in Fig. 4 and Supplementary Fig. 8, the level spacing, the superconducting gap energy and the transition temperature are extracted and plotted (Fig. 5a–c), respectively. Upon reducing the NC volume, both quantities display a sharp decrease to zero when the level spacing becomes of the order of the superconducting gap energy, ≃1 meV. See Supplementary Note 2 for a comparison with the results of Bose *et al*.^17^ on a system where the superconducting nanoparticles are strongly coupled to the normal substrate.

## Discussion

Figure 5 suggests that superconductivity disappears when the mean level spacing at the Fermi surface of the electron-type band (Fig. 5e), increases up to the superconducting gap energy. This is consistent with recent theoretical calculations^30^ and STM measurements^31^, which have shown that electron–phonon coupling is stronger for this electron-type band owing to its p–d character. Regarding the Bardeen-Cooper-Schrieffer ratio *k*_B_*T*_c_/Δ, within the experimental resolution, no significant deviation from the bulk value has been observed.

To summarize, we have found that a 2D electron gas of large Fermi wavelength constitutes an ideal substrate for studying Coulomb blockade in nanosized NCs evaporated in an ultra-high vacuum environment. This discovery leads us to observe, for the first time by STM, the parity effect and quantum confinement in isolated superconducting NCs and enabled the first demonstration of the Anderson criterion for the existence of superconductivity at single NC level. Furthermore, this new insight on the superconductor–InAs interface is of interest for topological superconductivity where Majorana islands are generated by depositing a superconductor on InAs nanowires^32^,^33^.

## Methods

### Relation between sample bias and energies

The Coulomb gap at zero bias results from Coulomb blockade that prevent charge fluctuations in the NC. As sketched in Supplementary Fig. 1, Coulomb blockade is lifted when the Fermi level of either one of the electrodes is aligned with one of the excited levels of the NC. Thus the amplitude of the Coulomb gap observed in the DC is given by 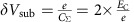, with 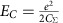.

The Coulomb peaks observed at higher voltages result from the shift of the electrochemical potential of the NC upon increasing the voltage bias across the double junction. This shift is given by:


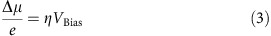


with:


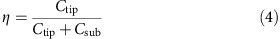


Charge states with increased number of electrons become accessible when the electrochemical potential changes by 2 × *E*_C_. Thus, the voltage difference between two charge states is given by:


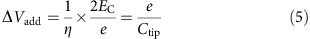


This formula shows that the addition voltage depends only on the capacitance *C*_tip_ and not on the capacitance *C*_sub_, as shown in Supplementary Fig. 1b, where a simulation of the conduction spectrum, using the Hanna and Tinkham model^26^ for two distinct values of the capacitance *C*_sub_.

### Addition energies

Following refs 1, 34, the total energy of a NC with *N* electrons is given by:


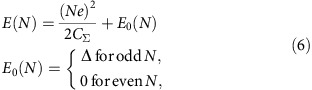


The electrochemical potential of a NC with an even (odd) *N* (*N*+1) number of electrons is given by:


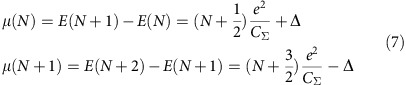


From these last equations, one obtains the addition energies for a NC with an even (odd) *N* (*N*+1) number of electrons:


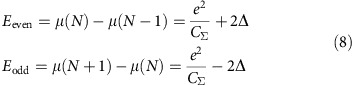


Thus, the difference of addition energies between two successive charge states is given by:


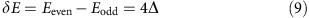


When the electronic spectrum of the NC is discrete, the level spacing *δ* should be included in the addition energy.


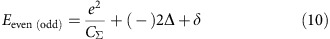


### Random level distribution

In metallic NCs, the electronic level distribution is described by RMT^29^,^35^. In a NC with strong spin–orbit coupling, RMT predicts that the level spacing should be described by a Gaussian symplectic ensemble. For this level distribution, shown in Supplementary Fig. 9, the width of the distribution, that is, the s.d., is equal to *σ*≃<*δ*> (refs 29, 36). Between two successive charge states, the addition energy can fluctuate by an amount of the order of *σ*, consequently, in average, the difference in addition energies between two successive charge states is given by:


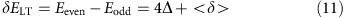


At temperatures above the superconducting transition temperature:


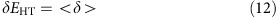


Thus, an estimation of the level spacing can be obtained by a measure of the difference in the addition energies above *T*_C_.

Furthermore, the gap amplitude can be obtained from:


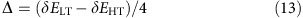


### Measurements details

The microscope used is a low temperature, *T*_base_=1.3 K, Joule–Thomson STM from SPECS accommodated with a preparation chamber operating in ultra-high vacuum at a base pressure *P*∼10^−10^ mbar. The DC curves d*I*/d*V* are measured with a standard lock-in procedure. An a.c. signal of amplitude ≃1 meV and frequency ∼777 Hz is employed.

### Data avaibility

The data that support the main findings of this study are available from the corresponding author upon request.

## Additional information

**How to cite this article:** Vlaic, S. *et al*. Superconducting parity effect across the Anderson limit. *Nat. Commun.* **8**, 14549 doi: 10.1038/ncomms14549 (2017).

**Publisher's note:** Springer Nature remains neutral with regard to jurisdictional claims in published maps and institutional affiliations.

## Supplementary Material

Supplementary Information Supplementary Figures 1-9, Supplementary Notes 1-2 and Supplementary References

## References

[b1] Averin D. V. & Nazarov Y. V. Single-electron charging of a superconducting island. Phys. Rev. Lett. 69, 1993–1996 (1992). 10046369 10.1103/PhysRevLett.69.1993

[b2] Lafarge P., Joyez P., Esteve D., Urbina C. & Devoret M. H. Two-electron quantization of the charge on a superconductor. Nature 365, 422–424 (1993).

[b3] Tuominen M. T., Hergenrother J. M., Tighe T. S. & Tinkham M. Experimental evidence for parity-based 2e periodicity in a superconducting single-electron tunneling transistor. Phys. Rev. Lett. 69, 1997–2000 (1992). 10046370 10.1103/PhysRevLett.69.1997

[b4] Eiles T., Martinis J. & Devoret M. Even-odd asymmetry of a superconductor revealed by the Coulomb blockade of Andreev reflection. Phys. Rev. Lett. 70, 1862–1865 (1993). 10053405 10.1103/PhysRevLett.70.1862

[b5] Lafarge P., Joyez P., Esteve D., Urbina C. & Devoret M. H. Measurement of the even-odd free-energy difference of an isolated superconductor. Phys. Rev. Lett. 70, 994–997 (1993). 10054257 10.1103/PhysRevLett.70.994

[b6] Higginbotham A. P. . Parity lifetime of bound states in a proximitized semiconductor nanowire. Nat. Phys. 11, 1017–1021 (2015).

[b7] Joyez P., Lafarge P., Filipe A., Esteve D. & Devoret M. H. Observation of parity-induced suppression of Josephson tunneling in the superconducting single electron transistor. Phys. Rev. Lett. 72, 2458–2461 (1994). 10055885 10.1103/PhysRevLett.72.2458

[b8] Aumentado J., Keller M. W., Martinis J. M. & Devoret M. H. Nonequilibrium quasiparticles and 2 e periodicity in single-cooper-pair transistors. Phys. Rev. Lett. 92, 066802 (2004). 14995261 10.1103/PhysRevLett.92.066802

[b9] van Woerkom D. J., Geresdi A. & Kouwenhoven L. P. One minute parity lifetime of a NbTiN Cooper-pair transistor. Nat. Phys. 11, 547–550 (2015).

[b10] Anderson P. Theory of dirty superconductors. J. Phys. Chem. Solids 11, 26–30 (1959).

[b11] Ralph D. C., Black C. T. & Tinkham M. Spectroscopic measurements of discrete electronic states in single metal particles. Phys. Rev. Lett. 74, 3241–3244 (1995). 10058147 10.1103/PhysRevLett.74.3241

[b12] von Delft J. & Ralph D. Spectroscopy of discrete energy levels in ultrasmall metallic grains. Phys. Rep. 345, 61–173 (2001).

[b13] Reich S., Leitus G., Popovitz-Biro R. & Schechter M. Magnetization of small lead particles. Phys. Rev. Lett. 91, 147001 (2003). 14611546 10.1103/PhysRevLett.91.147001

[b14] Zolotavin P. & Guyot-Sionnest P. Meissner effect in colloidal Pb nanoparticles. ACS Nano 4, 5599–5608 (2010). 20873723 10.1021/nn102009g

[b15] Savin A. M. . Parity effect in Al and Nb single electron transistors in a tunable environment. Appl. Phys. Lett. 91, 063512 (2007).

[b16] Hong I.-P., Brun C., Pivetta M., Patthey F. & Schneider W.-D. Coulomb blockade phenomena observed in supported metallic nanoislands. Front. Phys. 1, 1–8 (2013).

[b17] Bose S. . Observation of shell effects in superconducting nanoparticles of Sn. Nat. Mater. 9, 550–554 (2010). 20512156 10.1038/nmat2768

[b18] Brune H. Microscopic view of epitaxial metal growth: nucleation and aggregation. Surf. Sci. Rep. 31, 125–229 (1998).

[b19] Dombrowski R., Steinebach C., Wittneven C., Morgenstern M. & Wiesendanger R. Tip-induced band bending by scanning tunneling spectroscopy of the states of the tip-induced quantum dot on InAs(110). Phys. Rev. B 59, 8043–8048 (1999).

[b20] Tersoff J. Theory of semiconductor heterojunctions: the role of quantum dipoles. Phys. Rev. B 30, 4874–4877 (1984). 10.1103/physrevb.31.2526 9936077

[b21] Mönch W. Semiconductor Surfaces and Interfaces Springer (2001).

[b22] Morgenstern M. . Scanning tunneling microscopy of two-dimensional semiconductors: spin properties and disorder. Physica E 44, 1795–1814 (2012).

[b23] Su W. B. . Correlation between quantized electronic states and oscillatory thickness relaxations of 2D Pb Islands on Si(111)-(7 Ã—7) surfaces. Phys. Rev. Lett. 86, 5116–5119 (2001). 11384435 10.1103/PhysRevLett.86.5116

[b24] van Wees B. J. . Quantized conductance of point contacts in a two-dimensional electron gas. Phys. Rev. Lett. 60, 848–850 (1988). 10038668 10.1103/PhysRevLett.60.848

[b25] Pasquier C. . Quantum limitation on Coulomb blockade observed in a 2D electron system. Phys. Rev. Lett. 70, 69–72 (1993). 10053260 10.1103/PhysRevLett.70.69

[b26] Hanna A. E. & Tinkham M. Variation of the Coulomb staircase in a two-junction system by fractional electron charge. Phys. Rev. B 44, 5919–5922 (1991). 10.1103/physrevb.44.5919 9998444

[b27] Matveev K. A. Coulomb blockade at almost perfect transmission. Phys. Rev. B 51, 1743–1751 (1995). 10.1103/physrevb.51.1743 9978895

[b28] Aleiner I., Brouwer P. & Glazman L. Quantum effects in Coulomb blockade. Phys. Rep. 358, 309–440 (2002).

[b29] Alhassid Y. The statistical theory of quantum dots. Rev. Mod. Phys. 72, 895–968 (2000).

[b30] Floris A., Sanna A., Massidda S. & Gross E. K. U. Two-band superconductivity in Pb from *ab initio* calculations. Phys. Rev. B 75, 1–6 (2007).

[b31] Ruby M., Heinrich B. W., Pascual J. I. & Franke K. J. Experimental demonstration of a two-band superconducting state for lead using scanning tunneling spectroscopy. Phys. Rev. Lett. 114, 157001 (2015). 25933331 10.1103/PhysRevLett.114.157001

[b32] Das A. . Zero-bias peaks and splitting in an AlInAs nanowire topological superconductor as a signature of Majorana fermions. Nat. Phys. 8, 887–895 (2012).

[b33] Albrecht S. M. . Exponential protection of zero modes in Majorana Islands. Nature 531, 206–209 (2015). 10.1038/nature17162 26961654

[b34] Kouwenhoven L. P., Austing D. G. & Tarucha S. Few-electron quantum dots. Rep. Prog. Phys. 64, 701–736 (2001).

[b35] Halperin W. Quantum size effects in metal particles. Rev. Modern Phys. 58, 533–606 (1986).

[b36] Mehta M. L. Random Matrices Academic Press (2004).

