# Supplementary material for: Superconducting parity effect across the Anderson limit
Source: Nat Commun. 2017 Feb 27;8:14549. doi: 10.1038/ncomms14549 (PMC5333369; doi:10.1038/ncomms14549)
Supplement: Supplementary Information — Supplementary Figures 1-9, Supplementary Notes 1-2 and Supplementary References [file ncomms14549-s1.pdf]

Supplementary Figures

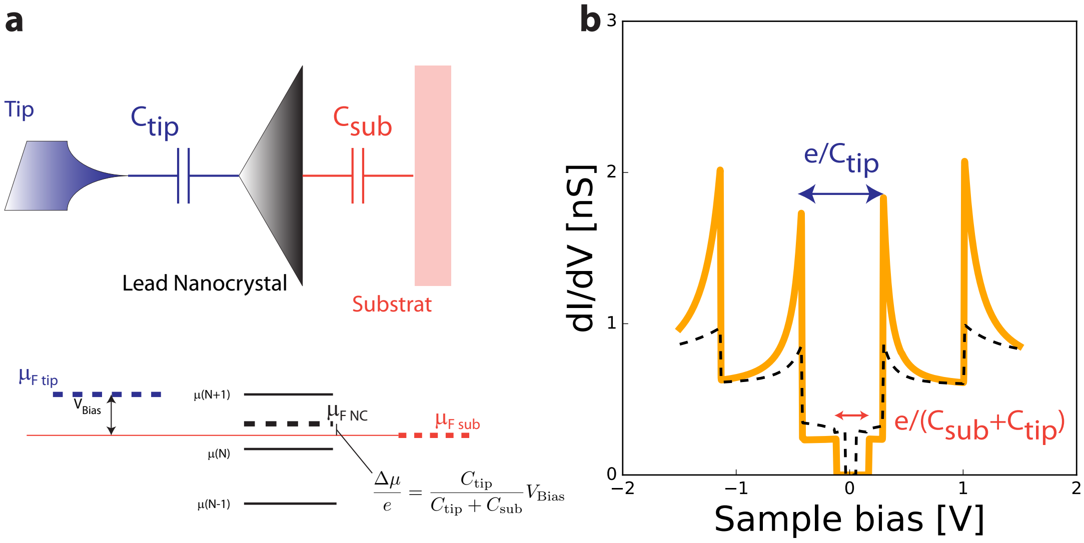

Supplementary Figure 1: **Electrostatic model.** (**a**) Schematic of the double junction Tip-Nanocrystal-Substrate. (**b**) Simulation of the conductance spectrum using Hanna and Tinkham model[1] for two distinct values of the capacitance C_{sub}, shown by the continuous and dash lines. The voltage interval between the Coulomb peaks do not change with the capacitance C_{sub}, only the amplitude of the Coulomb gap at zero bias changes, as indicated by the double-headed arrow.

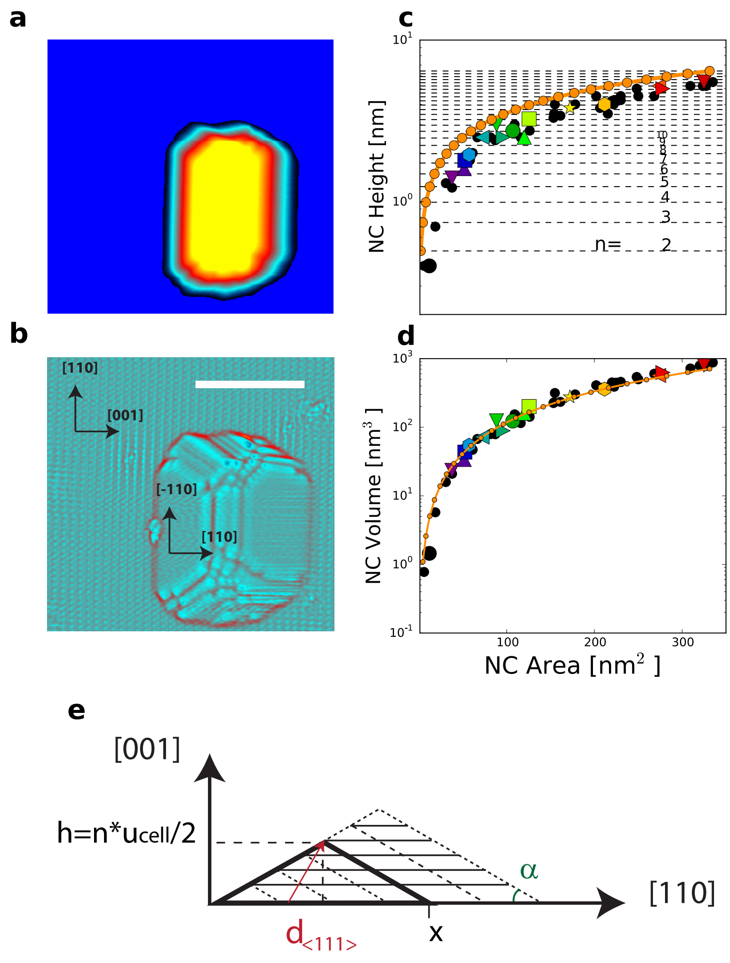

Supplementary Figure 2: **Structural model of the nanocrystals.** (**a**) Flooded image used to determine the surface and volume of the nanocrystal shown panel **b**. (**b**) Laplacian image of a nanocrystal. The scale bar is 8 nm. (**c**) The symbols show the experimental nanocrystal height as function of nanocrystal area. The orange dots show the calculated height as function of calculated area for pyramidal nanocrystals of increasing height where n is the number of atomic Pb rows. The horizontal dash lines indicate the height corresponding to the number of atomic rows n. (**d**) The symbols show the experimental nanocrystal volume as function of nanocrystal area. The orange dots show the calculated volume as function of calculated area for pyramidal nanocrystals of increasing height. (**e**) is a schematic of the model used to calculate the height, the area and the volume of the pyramidal nanocrystal.

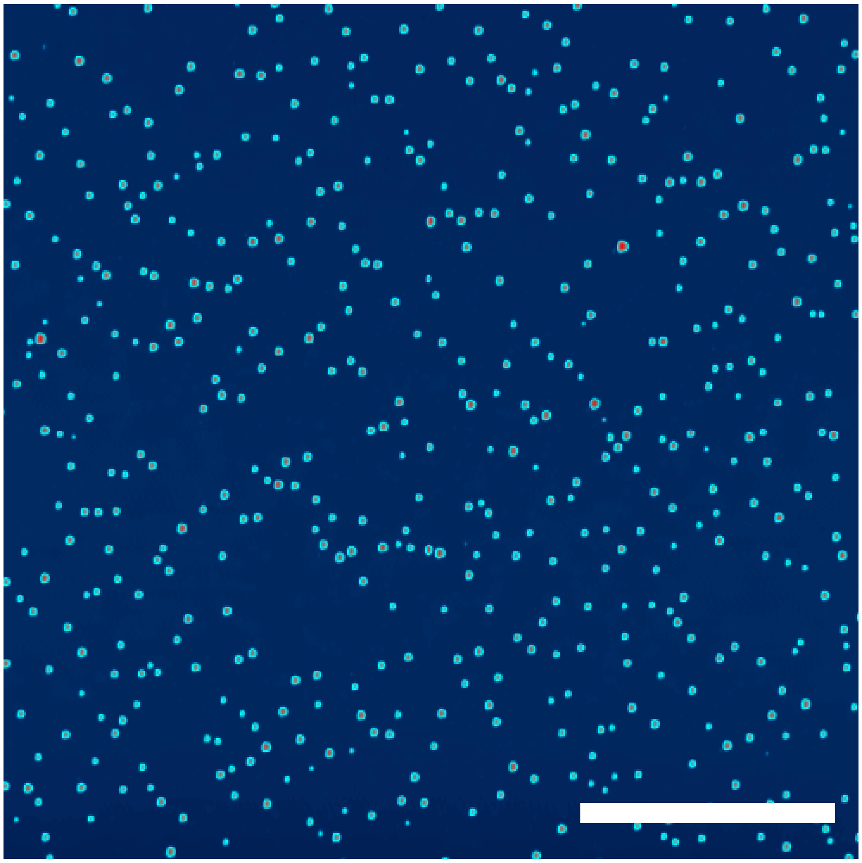

Supplementary Figure 3: **Topographic image of sample B.** Large size 1 μm$\times$1 μm topographic STM image (1 V, 30 pA) of Pb nanocrystals grown on the (110) InAs surface of sample B. The scale bar is 300 nm.

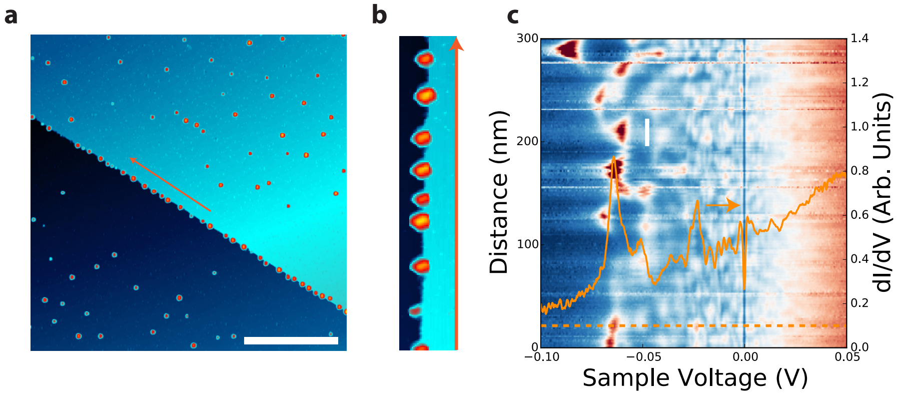

Supplementary Figure 4: **Fluctuations of the energy of the tip-induced QDot levels.** (**a**) Large topographic image ($1\mu m \times 1\mu m$) showing an atomic step edge against which the nanocrystals agglomerate. The scale bar is 300 nm. (**b**) Zoom on the area near the red arrow in panel **a** showing aligned nanocrystals along the atomic step edge. (**c**) Conductance dI/dV map as function of sample voltage and distance measured along the red arrow shown in panel **b**. The orange line is the conductance curve extracted from the map at the location indicated by the horizontal dash line. The map shows that the tip-induced QDot levels are fluctuating in energy because of the presence of nearby nanocrystals. These fluctuations are long range ($>$ 30 nm). The scale bar is 30 nm. This length is of the order of the Fermi wavelength of the 2D electron gaz. These fluctuations are the consequence of the changing electrostatic environment due to the random distribution of the Pb nanocrystals and dopants.

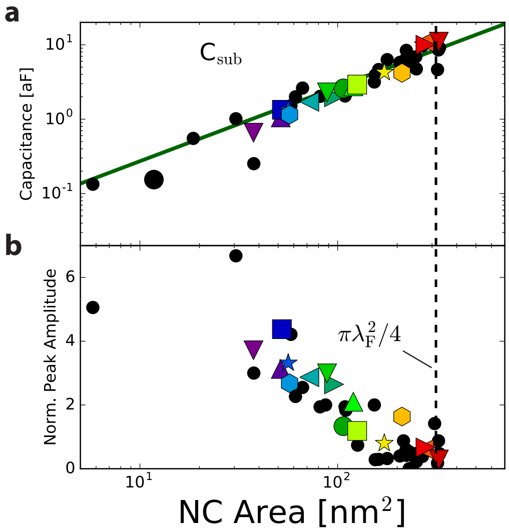

Supplementary Figure 5: **Substrate-nanocrystal capacitance on a large range.** (**a**) The capacitance C_{sub} extracted from the Coulomb gap at zero bias where the smallest nanocrystals have also been included. (**b**) The normalized Coulomb peak amplitude decreases when the nanocrystal area approach $\pi\lambda_F^2/4$.

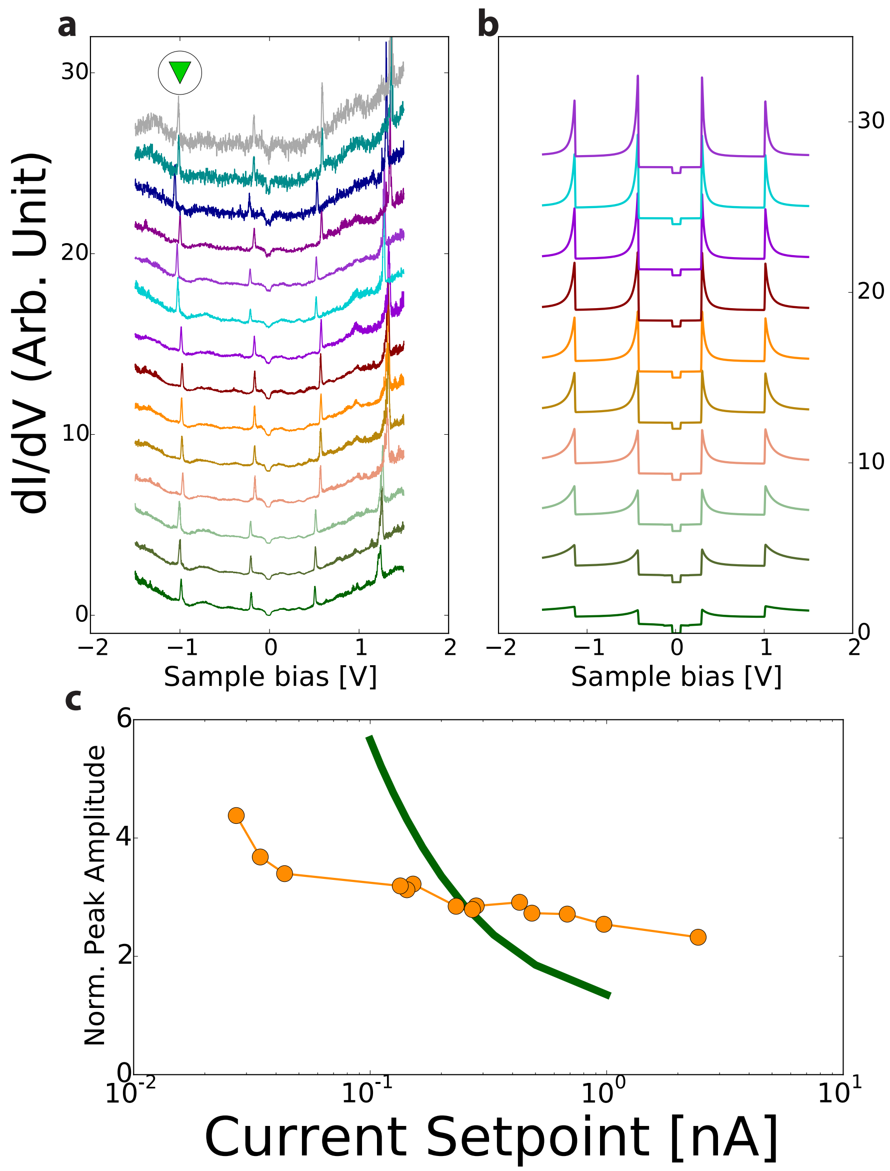

Supplementary Figure 6: **Coulomb peaks as function of tip height.** (**a**) Normalized and shifted DC curves, measured at different setpoints from I_{set}=5 nA (bottom) to I_{set}=30 pA (top). (**b**) Corresponding theoretical DC curves obtained from the weak coupling model, Ref. [1]. (**c**) Normalized peak amplitude measured experimentally (symbols) compared to the weak coupling model (line) of Hanna and Tinkham [1]. This model can describe qualitatively the evolution of the Coulomb peak amplitude with the current setpoint, i.e. the peak amplitude is the largest for the highest tunnel junction resistance. However, this model is not sufficient to describe quantitatively the evolution of the peak amplitude.

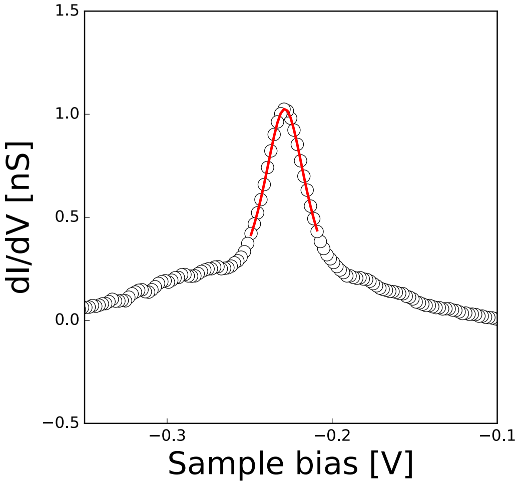

Supplementary Figure 7: **Lorentz fit of the Coulomb peak.** Zoom on a single Coulomb peak. The voltage position of the Coulomb peak maxima is obtained through a fit with a Lorentz function.

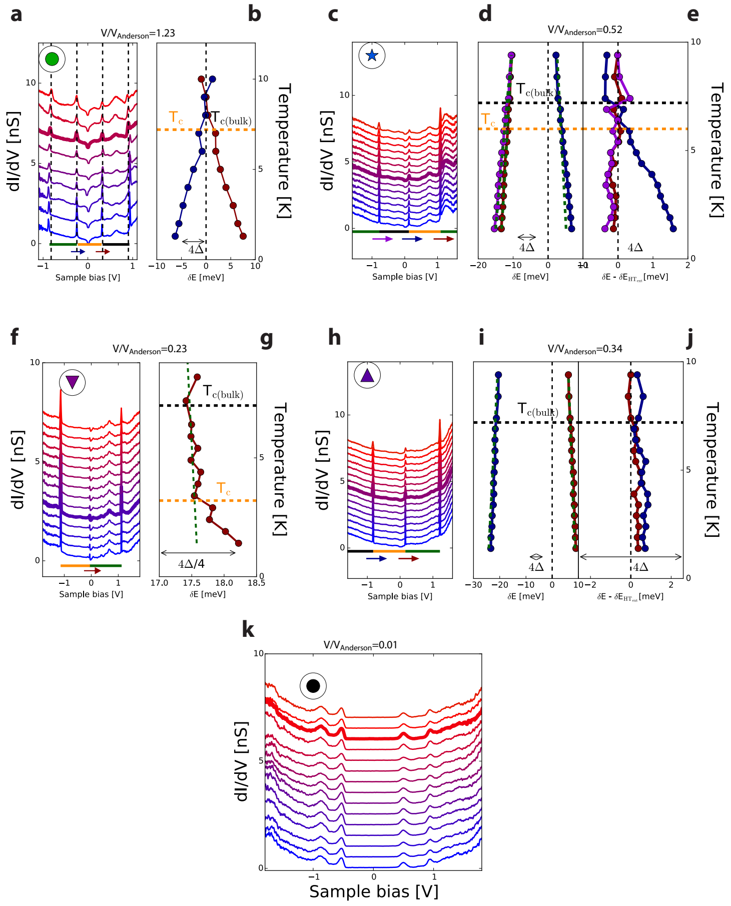

Supplementary Figure 8: **Conductance spectrum of additional nanocrystals** (**a**,**c**,**f**,**h**,**k**) DC curves for 5 nanocrystals of decreasing volume where V/V_{Anderson} is indicated on top of the panels. (**b**,**d**,**g**,**i**) Corresponding addition energies. The voltage separation between the Coulomb peaks is indicated by the horizontal bars of different colors. The difference in addition energies between two charge configurations is given by $\delta E = (\delta V_{\text{Head}} - \delta V_{\text{Tail}})/\eta$, where the head (tail) refers to the arrows shown in corresponding panels. (**e**,**j**) Difference $\delta E - \delta E_{\text{HT}_{\text{ext}}}$ where $\delta E_{\text{HT}_{\text{ext}}}$ is obtained from the extrapolation of δE at high temperature, shown as a dash green line. The $T_{\text{c(bulk)}}$ and energy gap Δ of bulk Pb are indicated in black. The extracted T_{c} is shown as an orange dash line. (**k**) In this very small nanocrystal, $V \simeq 0.01V_{\text{Anderson}}$, no Coulomb peaks are observed, only the large Coulomb gap at zero bias and quantum well states are observed.

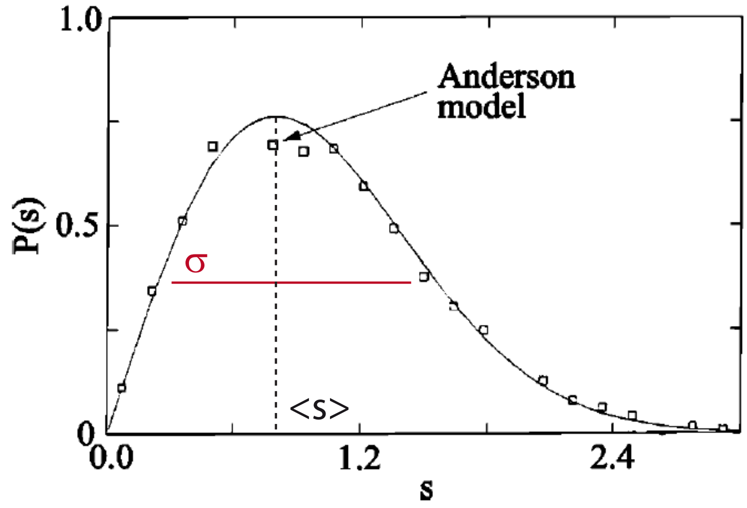

Supplementary Figure 9: **Random Matrix Distribution.** Distribution $P(s)$ of electronic levels as function of level separation s compared to the Anderson model, extracted from Ref. [2]. The width σ of the distribution is of the order of the level spacing.

Supplementary Notes

Supplementary Note 1. Structural model of the nanocrystals. The surface and the volume of the nanocrystals are obtained by a flooding method, Supplementary Fig. 2, as found in most STM images analysis softwares. The height of the nanocrystals and their volume are plotted Supplementary Fig. 2c and Supplementary Fig. 2d, respectively, as function of the nanocrystals area. As described in the main text, the nanocrystals facets are mostly oriented along the [111] direction. Thus, the shape of the nanocrystals is mostly pyramidal as sketched Supplementary Fig. 2e, where the [001] direction of Pb is oriented perpendicular to the substrate. For this geometry, the height of the nanocrystal is given by $h = n \times u_{\text{cell}}/2$ where u_{cell}=0.495 nm is the length of the unit cell of Pb; $u_{\text{cell}}/2$ is the distance between atomic rows along the [001] direction of Pb, which has a Face Centered Cubic (FCC) structure, and n is the number of atomic rows. Assuming that the nanocrystal is a perfect pyramid, its area can be calculated from the relation $s = x^2$ with $x = 2 \times h/\tan(\alpha)$, where the definition of α is given in Supplementary Fig. 2e. Furthermore, the volume of the pyramid can be calculated from $v = s \times h/3$. Plotting the calculated height as function of calculated area together with the experimental data, Supplementary Fig. 2d shows that this model can describe nicely the evolution of the nanocrystal height with the area of its base. Deviations from this model are expected since the nanocrystals are not perfect pyramids, their top are usually truncated. It can be noticed that the smallest nanocrystals measured are only two atomic rows high, i.e. one unit cell high. The spectra of these nanocrystals, shown Figure 3 (bottom curve) and Supplementary Fig. 8k, do not present any Coulomb peaks but only broad peaks due to the formation of quantum well states.

Supplementary Note 2. Comparison with the results of Bose et al.[3] In our work the nanoparticles are only weakly coupled to the conducting substrate as demonstrated by the presence of the Coulomb gap and the sharp Coulomb peaks. In the work of Bose et al.[3], no Coulomb gap or Coulomb peaks are observed, indicating that their nanoparticles are strongly coupled to the substrate. This is a fundamental difference between the two systems and has deep consequences on the evolution of the superconducting characteristics (T_c and Δ) with the size of the nanoparticle.

In our data, both quantities, T_c and Δ, do not change with the volume from 800 nm^3 down to the Anderson volume 100 nm^3. At the Anderson volume, both quantities go to zero very sharply. This suppression of superconductivity results from the suppression of pairing when the energy interval between two electronic levels becomes larger than the superconducting gap. This is the physical origin of the Anderson criterion, i.e. only levels within the superconducting gap energy form Cooper pairs.

In contrast, in the data of Bose et al., the superconducting gap value starts to decrease below a nanoparticle height about 10 nm, which corresponds to a volume of 2000 nm^3 for a semi-spherical shape, as indicated in their paper. Thus, in their system, the amplitude of the superconducting gap starts decreasing at a volume about 20 times larger than the Anderson volume. The authors attribute this reduction to quantum fluctuations of the superconducting order parameter. We could add, that, generally, the superconducting gap in strongly coupled normal-superconducting structures is expected to be smaller than the pure bulk superconductor because of the inverse proximity effect from the normal region onto the superconducting region.

The paper by Bose et al. also reports on the observation of the shell effect in Sn nanoparticles and the absence of this shell effect in the Pb nanoparticles. Within our experimental resolution, we did not observe the shell effect in the Pb nanoparticles either.

1. Hanna, A. E. & Tinkham, M. Variation of the Coulomb staircase in a two-junction system by fractional electron charge. *Physical Review B* **44**, 5919–5922 (1991).

2. Alhassid, Y. The statistical theory of quantum dots. *Reviews of Modern Physics* **72**, 895–968 (2000).

3. Bose, S. *et al.* Observation of shell effects in superconducting nanoparticles of Sn. *Nature materials* **9**, 550–554 (2010).
